# Films of Carbon Nanomaterials for Transparent Conductors

**DOI:** 10.3390/ma6062155

**Published:** 2013-05-27

**Authors:** Xinning Ho, Jun Wei

**Affiliations:** Singapore Institute of Manufacturing Technology, 71 Nanyang Drive, Singapore 638075; E-Mails: xnho@SIMTech.a-star.edu.sg (X.H.); jwei@SIMTech.a-star.edu.sg (J.W.); Tel.: +65-6793-8575 (J.W.); Fax: +65-6791-6377 (J.W.)

**Keywords:** transparent conductor, graphene, carbon nanotube, hybrid film, percolation, transfer printing, doping, grain boundaries, contact resistance

## Abstract

The demand for transparent conductors is expected to grow rapidly as electronic devices, such as touch screens, displays, solid state lighting and photovoltaics become ubiquitous in our lives. Doped metal oxides, especially indium tin oxide, are the commonly used materials for transparent conductors. As there are some drawbacks to this class of materials, exploration of alternative materials has been conducted. There is an interest in films of carbon nanomaterials such as, carbon nanotubes and graphene as they exhibit outstanding properties. This article reviews the synthesis and assembly of these films and their post-treatment. These processes determine the film performance and understanding of this platform will be useful for future work to improve the film performance.

## 1. Introduction

As electronic devices, such as touch screens, displays, solid state lighting and photovoltaic devices become more prevalent in our lives, the demand for transparent conductors increases. Doped metal oxides, especially indium tin oxide (ITO) [1,2] are often used as transparent conductors. They exhibit low electrical sheet resistance (<10 Ω/sq) and high optical transparency (>80%). The production of ITO films can also be easily scaled up. However, the material suffers several drawbacks. As ITO is very brittle and can fracture at low strains [3], it cannot be integrated into flexible devices, which is an area many semiconductor giants are looking to develop. Furthermore, due to the increasing scarcity of indium, a component material of ITO, the production of ITO may become very expensive. Hence, there is an interest in the exploration of alternative materials. These materials include conducting polymers [4,5,6], metal nanowires [7,8,9,10], thin metal films [11,12] and carbon nanomaterials [13,14,15,16,17,18,19,20,21,22,23]. 

Conducting polymers have exhibited good electrical, optical and mechanical properties [4]. However, they suffer from electrical instability. Exposure to environmental elements like humidity, high temperature or UV light deteriorates the electrical conductivity [5,6]. Alternative materials based on metals also face similar challenges. Although metal nanowires and thin metal films display superb electrical conductivity intrinsically, they oxidize easily and their electrical conductivity degrades accordingly [7,8,9,10,11,12]. Films based on carbon nanomaterials, such as carbon nanotube (CNT) and graphene have been of particular interest due to their good electrical, optical and mechanical properties, as well as good chemical stability. They have been found to exhibit low sheet resistance, high optical transparency, good flexibility and stability over time. Hence, they are appealing in novel applications requiring flexibility. This article reviews the synthesis, assembly and post-treatment of carbon nanotube (CNT), graphene and carbon based hybrid films and their impact on the film performance which can provide insights on opportunities for future work to improve the film performance.

## 2. Carbon Nanotube (CNT) Films

Carbon nanotube (CNT), a one-dimensional material, is extensively studied by various groups due to its attractive properties. CNT exhibits low electrical resistivity [24,25] and high current carrying capability [26], making it an ideal material for electrical conductors. A nanometer-sized CNT is also extremely small, which renders it transparent. Hence, a thin film of CNTs has emerged as a promising material for transparent conductors [13,14,15,16,17,18]. Besides good electrical conductivity and transparency, CNTs demonstrate exceptional mechanical properties. The fracture strain is up to 30% [27], which makes it ideal for use in flexible electronics. Despite the impressive properties exhibited by individual CNT, the use of an individual CNT is not practical for real world applications, which often require higher current output. The obvious solution to which is the use of a CNT film. However, such films suffer from poorer electrical conductivity and transparency than individual CNTs. In this section, we examine the factors, which degrade the CNT film performance to provide insights for improving the film properties as a transparent conductor. 

### 2.1. Properties of Carbon Nanotube (CNT) Thin Films

Various techniques have been developed to synthesize CNTs. Some common techniques include arc discharge [28], laser ablation [29] and chemical vapor deposition [30]. CNTs synthesized via arc discharge and laser ablation are often dispersed in solution to form CNT ink, which can be deposited on various substrates by different methods to form CNT network films. Solution processed CNT films are very attractive for large area and low cost commercial applications. 

Carbon nanotubes (CNTs) can be dispersed in solution via three main ways: dispersion in organic solvents [31], dispersion in aqueous media via dispersing agents like surfactants [32,33,34,35] and dispersion in solution by functionalizing the CNTs [36,37]. Direct dispersion of CNTs in organic solvent is a simple and straightforward method. However, CNTs can only be dispersed in low concentration in organic solvents, which is not practical for commercial applications. Use of dispersing agents like amphiphilic surfactants, assists the dispersion of hydrophobic CNTs in aqueous media like water. The hydrophobic interaction between the tails of the amphiphilic surfactants and the CNTs is very strong. A monolayer of surfactants is adsorbed on the CNT walls and a stable carbon nanotube-surfactant monolayer micelle is formed [32]. This allows CNTs to be dispersed in higher concentration in aqueous media. The presence of insulating surfactants in deposited CNT films formed from such solution decreases the electrical conductivity of the film. Hence, removal of surfactants after deposition is essential [38]. The last dispersion technique of functionalizing CNTs facilitates the dispersion of CNTs in solution by increasing the attraction between the CNTs and the solvent. Acid treatment of CNTs introduces carboxylic acid group (COOH) to the CNTs, which assists the dispersion [36]. However, excessive functionalization also introduces defects on the CNT structure, which degrades the electrical conductivity [37]. 

**Figure 1:**
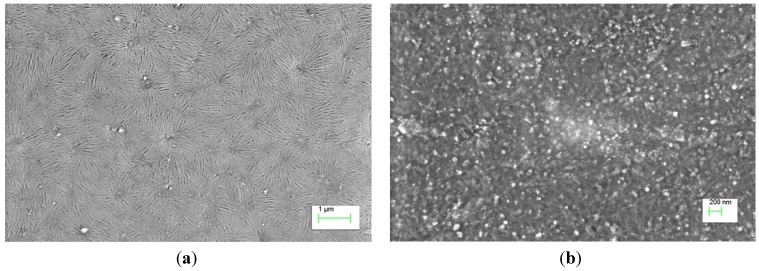
Scanning electron microscope (SEM) images of (**a**) “clean”; and (**b**) “dirty” carbon nanotube (CNT) network films formed from solution. Many impurity particles which are present in the “dirty” CNT network films are absent in the “clean” CNT network films.

Carbon nanotubes (CNTs) are often dispersed in solution by ultrasonication. This step is essential as it breaks down the CNT aggregates to disperse them in the solvent. As the process is very harsh, defects are introduced, which can degrade the electrical conductivity of the CNT [39]. Besides introducing defects, sonication also shortens the tube length [40], which has adverse effects on the electrical conductivity of a CNT film, which will be discussed in detail in a subsequent section. The presence of impurities from the synthesis process, such as catalyst particles and amorphous carbon also degrades the electrical properties of the CNT film formed from solution. Parts a and b of Figure 1 show scanning electron microscope (SEM) images of “clean” and “dirty” network CNT films formed from solution respectively. The “clean” film has a relatively lower concentration of impurities on the film while huge particles are visibly present on the “dirty” film over large areas. 

Carbon nanotube (CNT) films formed directly via chemical vapor deposition (CVD) [41,42] generally exhibit higher structural perfection, longer tube lengths and higher purity than solution processed CNT films. Hence, CVD grown films tend to be of higher quality. However, solution processed films can potentially be processed by low cost and large scale production so more efforts have been focused on solution processed films for commercial applications. 

Another important factor that determines the carbon nanotube (CNT) film property is the characteristics of individual CNTs. Synthesized CNTs are not uniform in conductivity type, length and diameter. Longer and larger diameter metallic CNTs are more desirable for transparent conductor applications. 

**Figure 2:**
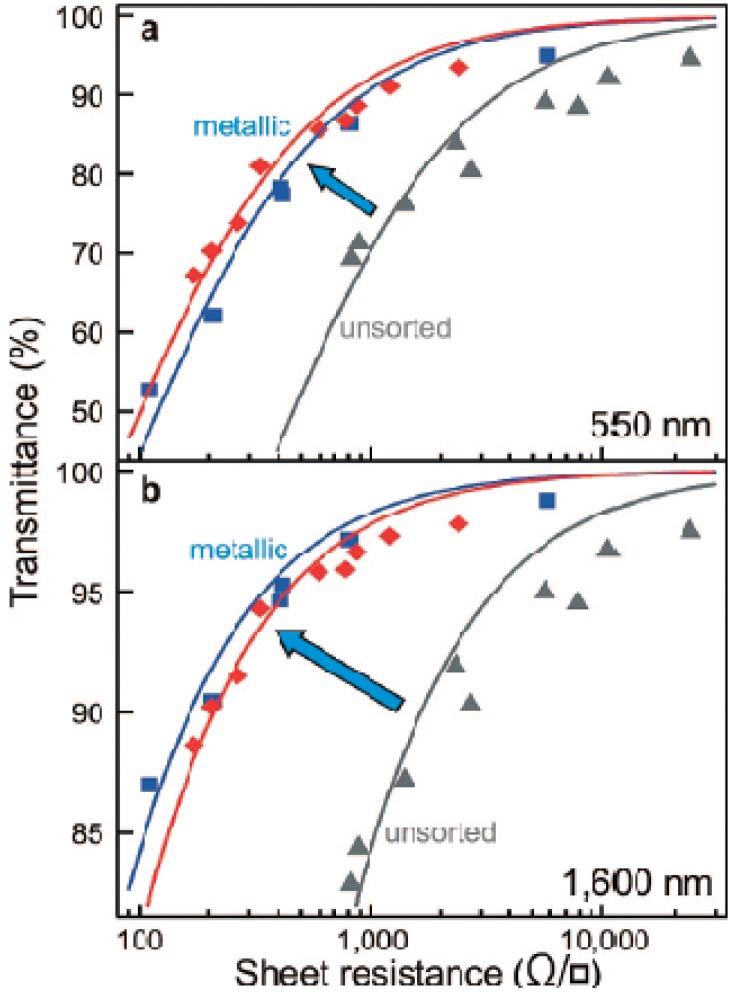
Transmittance versus sheet resistance of transparent conductive films generated from CNTs at (**a**) 550 nm; and (**b**) 1600 nm wavelengths. The materials used were metallic CNTs with principal diameters of 0.9 nm (red diamond symbols), 1.0 nm (blue square symbols) and a mixture of metallic and semiconducting CNTs (gray triangle symbols). Reprinted with permission from [48]. Copyright (2008) by the American Chemical Society.

The lower resistivity of metallic carbon nanotubes (CNTs) relative to semiconducting CNTs is attributed to the longer mean free path in metallic CNTs. Ballistic conduction in metallic CNTs can span up to micrometer range but semiconducting CNTs have a series of barriers to conduction along their lengths [43,44]. Resistivity of metallic CNTs have been found experimentally to be between 6 and 30 kΩ/µm [45,46,47] while semiconducting CNTs have higher resistivity, which is dependent on the gate voltage when used as the semiconducting channel in a transistor [45]. In fact, films based on solely metallic CNTs have demonstrated lower sheet resistance at a fixed transparency than films based on a mixture of metallic and semiconducting CNTs (Figure 2) [48]. It is also evident from Figure 2 that a CNT film with lower sheet resistance has lower transparency. The transparency of CNT films is dominated by the absorption of CNTs in the film. Hence, when a thicker CNT film (with higher density of CNTs) is prepared, there is more absorption, which results in a lower transparency. However, the higher density of CNTs in the film also results in an improvement in the electrical conductivity by increasing the number of electrical pathways. Therefore, control of the density of CNTs in the film is essential for optimizing the sheet resistance and transparency of the films.

Besides the lower sheet resistivity of metallic CNTs, the improvement in electrical conductivity of a film based on metallic CNTs only is attributed to the lower junction resistance between two metallic CNTs compared to junction resistance between one metallic CNT and one semiconducting CNT [49]. The junction resistance between two metallic CNTs or two semiconducting CNTs is low as there is a finite density of states at the junction for tunneling on either side of the junction. On the other hand, the junction resistance between a metallic CNT and semiconducting CNT is high as a Schottky barrier forms due to charge transfer from the metallic CNT to the semiconducting CNT [49]. 

Hence, sorting of carbon nanotubes (CNTs) or selective synthesis of metallic CNTs is very crucial. Various groups have explored different means of sorting or selective synthesis, such as selective chemical functionalization [50,51], selective electrical breakdown [52], density differentiation [53,54] and dielectrophoresis [55] with varying degrees of success. However, these methods are tedious and the yield is low, making it unsuitable for low cost commercial applications.

Larger diameter CNTs are preferred for transparent conductors because they carry more current than smaller diameter CNTs [56,57]. In fact, the peak mobility of CNT is found to scale with the square of the CNT diameter and the maximum conductance scales linearly with the CNT diameter [56]. Various groups have explored means to grow CNTs with selective CNT diameters [48,53,58,59,60,61]. Hersam group has been successful in achieving monodispersed CNTs via density differentiation [48]. However, such CNT films are colored and non-ideal as transparent conductors because CNTs with a monodisperse bandgap have a narrow range of absorption peaks. 

**Figure 3:**
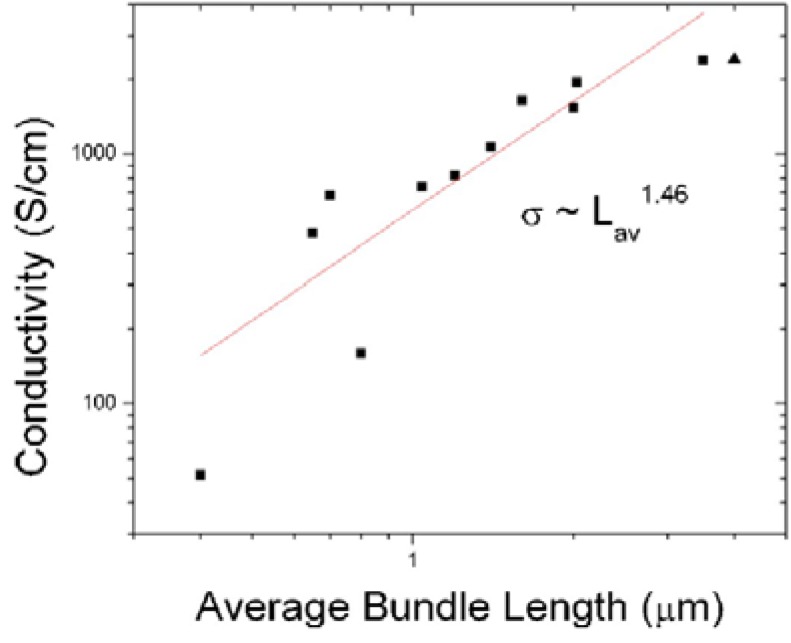
Variation of the CNT network electrical conductivity with the average bundle length of the CNTs in the network. The relationship between the conductivity of the CNT network and the average bundle length is σ ~ L_av_^1.46^. Reprinted with permission from [62], Copyright (2006) by the American Institute of Physics.

Another important factor that influences the conductivity of the CNT film is the tube length of the CNTs [62,63]. The conductivity of CNT networks, σ, varies as σ ~ L_av_^1.46^ [62] (Figure 3). This is expected as the junction resistance between CNTs is higher than the intrinsic tube resistance of the CNTs. The resistance between two metallic CNTs or two semiconducting CNTs is between 200 and 400 kΩ and about 100 times smaller than the junction resistance between a metallic CNT and a semiconducting CNT. On the other hand, the tube resistivity of CNTs is between 6 and 30 kΩ/µm. As the tube length in the network approaches 20 µm, the intrinsic tube resistance is comparable to the junction resistance. Hence, beyond 20 µm, there is limited improvement in conductivity of the network with an increase in tube length. 

Sheet resistance (R_s_) and transmittance (T) of carbon nanotube network are related by

(1) $$T(\lambda )={\left(1+\frac{188.5}{R_{s}}\frac{\sigma_{Op}(\lambda )}{\sigma_{DC}}\right)}^{-2}$$

where σ_DC_ and σ_Op_ are the electrical and optical conductivities respectively [16]. Hence, high σ_DC_/σ_Op_ is desired for a low sheet resistance and high transparency network. The electrical conductivity, σ_DC_, is found to correlate with the network morphology. The mean diameter of the CNT bundles (bundled due to agglomeration of solution processed CNTs) has an inverse relationship with σ_DC_. This is because the smaller the bundle, the higher the junction density and number of electrical pathways in the network. Hence, debundling of CNTs is desirable [16].

The properties of CNTs in the CNT network film are examined in this section. Long and large diameter metallic carbon nanotubes, which are debundled are most desirable for transparent conductor applications.

### 2.2. Carbon Nanotube (CNT) Film Assembly

Besides the properties of CNTs in the CNT network film, the way that the CNT film is assembled is also very crucial to the electrical and optical properties of the film. There are various methods to deposit solution based CNTs onto a receiving substrate, such as vacuum filtration followed by transfer printing [15,64], spray coating [65,66] and controlled flocculation [67,68]. Transfer printing is often used to transfer chemical vapor deposition (CVD) grown CNT films from the growth substrate to another receiving substrate [69]. 

The vacuum filtration method is one of the most commonly used methods to assemble solution deposited CNT films. The process is simple and straightforward, and yields a uniform film, which is desired for many transparent conductor applications such as in photovoltaic cells and light emitting diodes. A uniform film with high surface roughness can potentially cause short circuit in thin film devices. However, the vacuum filtration method is limited in scale as it is determined by the size of the membrane filter. 

Spray coating is another commonly used method. It is very versatile and can be used to coat surfaces with various shapes and curvatures. However, care must be taken to prevent re-agglomeration of the CNTs as they are deposited on the heated surface [66]. Re-agglomeration results in bundled CNTs, which decreases the electrical conductivity of the deposited film (as discussed in the earlier section). Careful selection of the surfactant used in the CNT solution and the parameters of spraying can mitigate this problem [66]. 

Controlled flocculation, another method to assemble solution based CNTs, deposits CNTs by adding liquids that are miscible with the suspending solvent and interact well with the surfactant. This drives the CNTs out and deposits them on the desired substrate. Methanol is often used [67,68]. In this case, re-agglomeration, which results in bundling is also an issue so the process must occur close to the surface of the receiving substrate to minimize agglomeration before deposition [67].

Finally, transfer printing of chemical vapor deposition (CVD) grown CNTs has proven to be an effective method to transfer CVD grown CNTs. The method is deterministic and the CNTs can be completely transferred from a donor substrate to a receiving substrate [69]. It is evident from this section that the assembly method of CNTs is very important and can impact the electrical conductivity of the film obtained. Hence, careful optimization of the assembly method is essential to yield high quality films.

### 2.3. Post Treatment 

After the assembly of the CNT film, post deposition treatment is often carried out to improve the electrical conductivity by improving the junction resistance between two CNTs [70,71], removal of the insulating surfactants [72,73] and doping of the CNTs [74,75,76,77,78,79].

**Figure 4:**
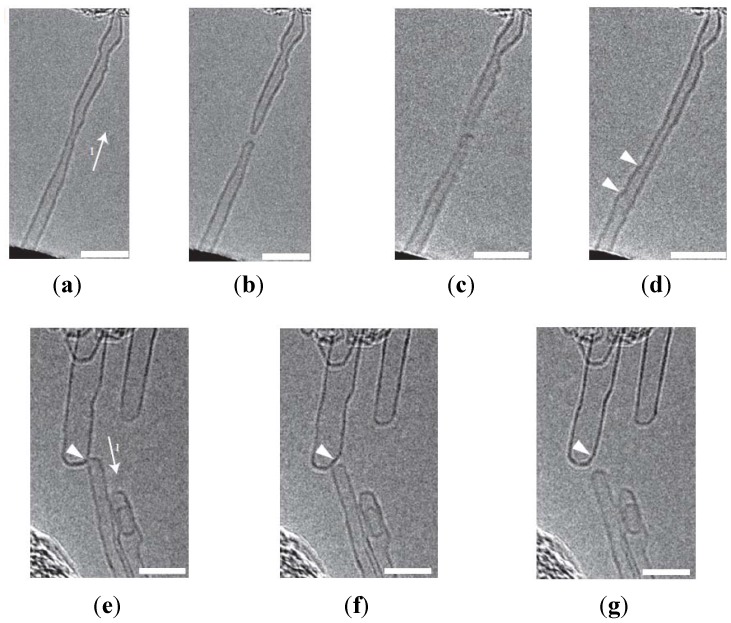
Joining of two CNTs with similar diameter works but fails for two CNTs with different diameters. (**a**–**d**) Successful joining of a CNT split into two separate CNTs by electrical breakdown (**a**,**b**). After contacting the two CNTs and applying a large voltage (**c**), the two CNTs bond and a new CNT forms (**d**); (**e**–**g**) Unsuccessful joining of two CNTs with different diameters. Scale bars are 5 nm in all parts of the Figure. Reprinted with permission from [70]. Copyright (2008) by the Nature Publishing Group.

The junction resistance between two CNTs is expected to decrease when they are joined. Two CNTs can be joined by Joule heating [70]. By contacting two CNTs and applying a large voltage across them, current flows across the two CNTs and electromigration occurs. Joining is only possible between CNTs of similar diameter as shown in Figure 4. Hence, this method is not feasible for CNT networks with a large range of diameters. Another way to join CNTs is via electron irradiation in a scanning electron microscope (SEM) [71]. By focusing the electron beam at the junction, carbon contamination is deposited selectively at the junction. The graphitic material, which connects two CNTs is expected to be electrically conductive. However, the two methods mentioned above are not scalable and will not be useful for large scale commercial use.

The electrical conductivity of CNT network can also be improved by removing surfactants often used to disperse CNTs in solution. Some surfactant residues are deposited together with CNTs during CNT film assembly. As the surfactant is insulating, the electrical conductivity of the CNT film is compromised. Hence, after CNT film assembly, removal of the surfactant improves the electrical conductivity. Surfactant can be removed by washing with water, followed by acetone [72] or by acid treatment, such as immersion in HNO_3_ [73].

Another commonly used method to improve the electrical conductivity of CNT network is by doping. Various types of dopants have been studied and found to improve the electrical conductivity by different extents. Doping of CNTs by vapor phase reactions with bromine (electron acceptor) or potassium (electron donor) has yielded decreased resistivity by a factor of 30 [74]. Doping by NO_2_ shifts the Fermi level closer to the valence band and conductivity improves [75]. P doping by dopants, such as HNO_3_, SOCl_2_ or I_2_ also prove effective [76,77]. Another commonly used p-type dopant is tetracyanoquinodimethane (TCNQ) [78,79]. However, all these methods suffer from a stability problem. The dopants desorb and electrical conductivity degrades with time. More work remains to find an alternative dopant. The successful commercialization of CNT transparent conductors is dependent upon further improvement of the electrical and optical properties, scalability, reproducibility and cost effectiveness of the production.

## 3. Graphene Films

Graphene, the two-dimensional allotrope of carbon, has generated much interest because of its high mobility [80], transparency [81] and flexibility [21,82]. Besides near ballistic transport in suspended graphene [80], the transmittance through a single layer of graphene is extremely high (97.7%) [81]. Graphene has also demonstrated extreme flexibility [21]. These outstanding properties of graphene can lead to its potential application as a flexible transparent conductor. Nonetheless, challenges remain for the material before its successful application in the real-world. Large scale production of low sheet resistance and high optical transparency graphene films that are electrically stable over time has yet to be established. In this section, we review the factors influencing the graphene film performance, in particular the synthesis, assembly and post-treatment of graphene films.

### 3.1. Synthesis and Assembly of Graphene Films

#### 3.1.1. Solution Processed Films

Solution processed graphene films have been studied extensively because their production via roll-to-roll processing can potentially be scaled up for commercial applications. There are two main forms of precursors to graphene in solution: graphitic precursors [83,84,85,86] or graphite oxide precursors [82,87,88,89,90,91,92,93,94,95,96].

As graphite is hydrophobic, surfactants are often used to assist the graphite to disperse in organic solvents. Lotya *et al*. [85] demonstrated that the surfactant, sodium dodecylbenzene sulfonate (SDBS), aids the dispersion of graphite in water. The ionic surfactant adsorbs onto the graphite flakes and prevents the re-aggregation of graphite flakes suspended in water via Coulomb repulsion. Hence, a large percentage of flakes have less than five layers while ~3% of the flakes are monolayer. The graphite flakes are also found to be largely free of defects or oxides, which improves the electrical conductivity of the flakes. However, the flake dimensions (*i.e*., the width and length) tend to be <400 nm, which will increase the number of inter-flake interfaces when a large surface area film is formed from such dispersions. The large number of inter-flake interfaces present will decrease the electrical conductivity of the film formed. Thin films can be formed from graphite dispersions via various techniques like vacuum filtration [83,85], spray coating [83,85] or Langmuir-Blodgett assembly [84]. 

**Figure 5:**
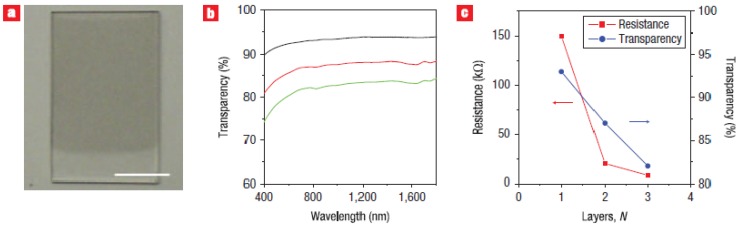
Langmuir-Blodgett (LB) films of graphene sheet. (**a**) A photograph of a film of graphene sheet deposited on the top portion of a quartz substrate. The scale bar is 10 mm; (**b**) Transmission spectra of the single layer (black curve), double layer (red curve) and triple layer (green curve) LB films; (**c**) Sheet resistance (red curve) and transparency at a wavelength of 1000 nm (blue curve) of the LB films with different number of layers. Reprinted with permission from [84]. Copyright (2008) by the Macmillan Publishers Limited.

Another group [84] reported dispersing graphite in dimethylformamide (DMF) with the aid of the surfactant, 1,2-distearoyl-sn-glycero-3-phosphoethanolamine-N-[methoxy(polyethyleneglycol)-5000] (DSPE-mPEG). Thin films formed from this dispersion via the Langmuir-Blodgett (LB) assembly method demonstrate a sheet resistance down to 8 kΩ/sq and transparency up to 93%, depending on the number of LB films deposited, as shown in Figure 5. Many challenges still remain as most applications for transparent conductors, such as touch screens require a sheet resistance <500 Ω/sq and transparency >85% [96].

Another form of precursor to graphene in solution is graphite oxide precursors. By oxidizing graphite via the Hummers method [97] or a modified Hummers method [98,99], the graphite oxide formed can now be dispersed in water. The addition of surfactant is no longer necessary. Graphite oxide films can be formed from the graphite oxide dispersion via similar methods to the graphite dispersions. Other methods of film assembly include spin coating [88] and dip coating [94]. However, upon film formation, the graphite oxide film has to be reduced to form graphene or graphite film. The graphite oxide film can be reduced via thermal annealing or chemical methods. 

**Figure 6:**
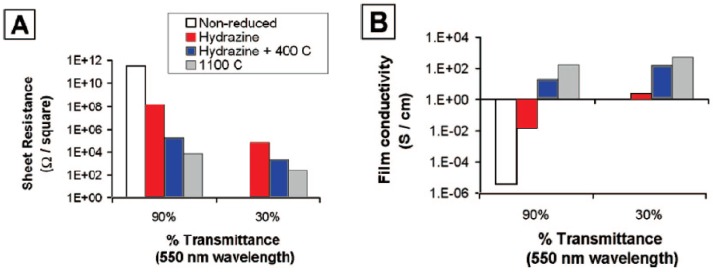
Electrical properties of reduced graphene oxide films that have been treated with different reduction methods. (**A**) Sheet resistance of the films with either 90% or 30% transmittance at a wavelength of 550 nm; (**B**) Film conductivity of the films with either 90% or 30% transmittance at a wavelength of 550 nm. Reprinted with permission from [88]. Copyright (2008) by the American Chemical Society.

**Figure 7:**
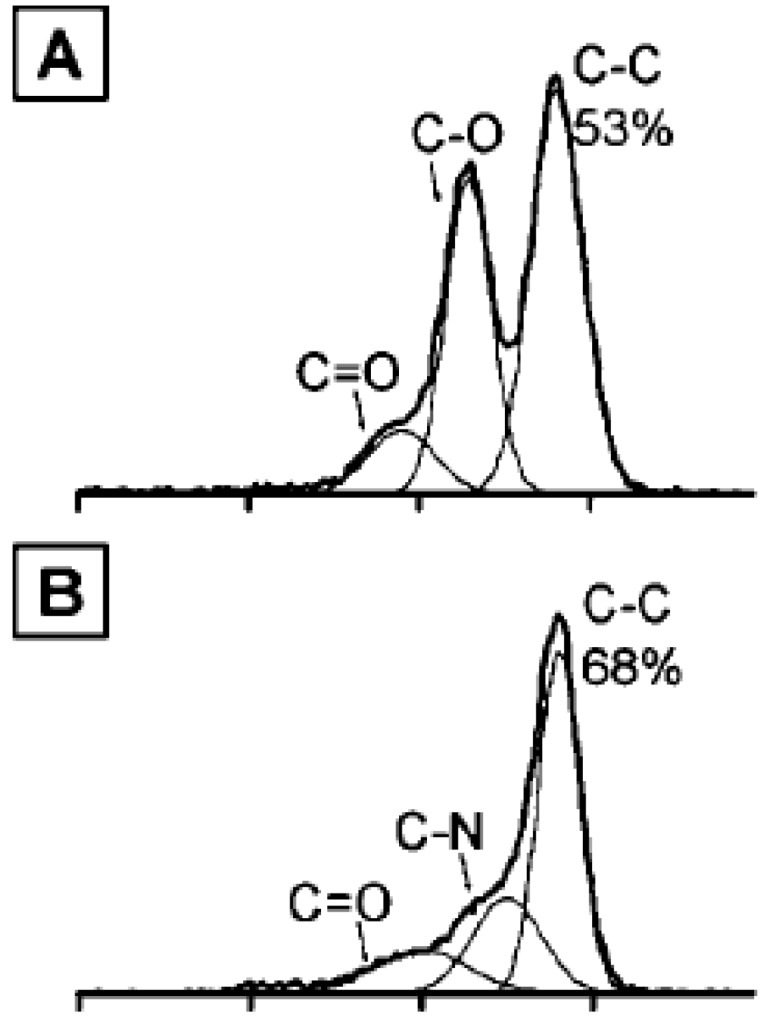

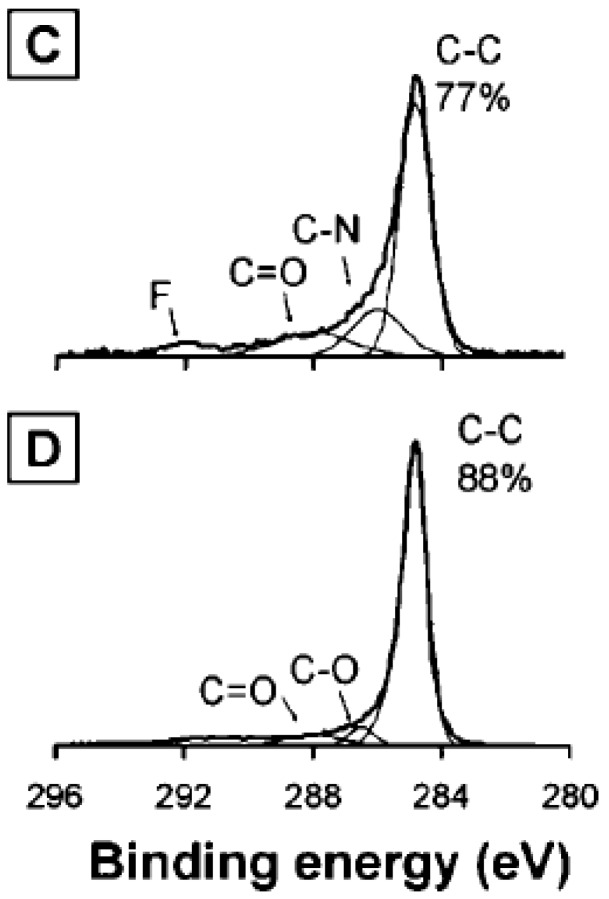
XPS analysis of the reduced graphene oxide films after different reduction treatments. **(A)** Non-reduced film; **(B)** Hydrazine-reduced film; **(C)** Film reduced by hydrazine and annealing at 400 °C; **(D)** Film reduced by annealing at 1100 °C. Reprinted with permission from [88]. Copyright (2008) by the American Chemical Society.

Becerril *et al*. compared the sheet resistance and transparency of films formed via various reduction treatments [88]. Three treatment methods are examined: reduction by hydrazine vapor, reduction by hydrazine vapor and annealing at 400 °C under argon flow and annealing at 1100 °C in vacuum. Figure 6 shows the outcome of his study. Thermal annealing at 1100 °C proves most effective while reduction via hydrazine vapor is the least effective. Characterization of the films after reduction using X-ray photoelectron spectroscopy (XPS) explains the phenomenon, as shown in Figure 7. Hydrazine treatment incorporates nitrogen into the samples by partially reducing the carbonyl functionalities to hydrazone groups. This decreases the relative content of carbon unbounded to oxygen or nitrogen and decreases the film conductivity of the sample. Annealing at 400 °C can desorb some of the nitrogen, leading to an improvement in film conductivity. Hydrazone groups are absent in samples that are annealed at 1100 °C and these samples show the best electrical conductivity. Complete reduction of graphene oxide films is very crucial as it has been found that lattice vacancies that cannot be healed during reduction can result in a three orders of magnitude decrease in electrical conductivity for such films relative to graphene film [95]. 

Mattevi *et al*. also reported that incomplete reduction has detrimental effects on the electrical properties of reduced graphene films [100]. The residual oxygen in the graphene oxide film forms sp^3^ bonds with the carbon atoms in the basal plane. The sp^3^ bonds disrupt the transport of charge carriers delocalized in a sp^2^ network. Hence, the electrical conductivity is lower in a partially reduced film relative to a completely reduced film. 

**Figure 8:**
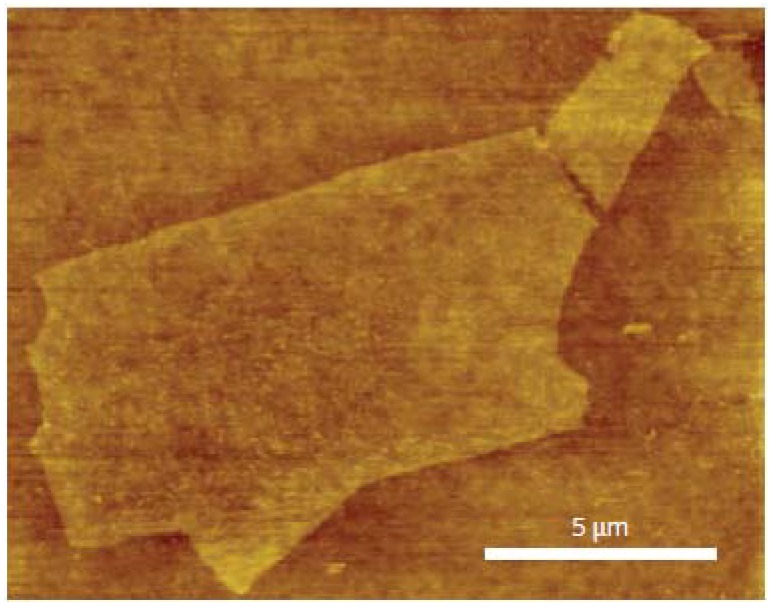
Atomic force microscope (AFM) image of a large scale graphene. Reprinted with permission from [87]. Copyright (2009) by the Macmillan Publishers Limited.

Besides complete reduction of graphene oxide films, large graphene sheets are desired as they decrease the inter-flake interfaces. Large-scale graphene sheets with an area up to 20 × 40 µm have been demonstrated by Tung *et al*. [87] as shown in Figure 8. By dispersing graphene oxide in pure hydrazine, hydrazinium graphene dispersions are formed. A film can be formed from the dispersion via spin-coating.

The main advantage of solution processed films is their compatibility with large scale and low cost roll-to-roll processing. The key disadvantages are the incomplete reduction of graphene oxide and the small dimensions of the graphene sheets formed. Development of methods to overcome these issues will result in films with better electrical conductivity, which is an important figure of merit for transparent conductors.

#### 3.1.2. Chemical Vapor Deposition (CVD) Growth Films

Graphene films can also be formed by chemical vapor deposition (CVD) growth. The process usually involves breaking down a gaseous carbon feedstock (e.g., methane) in hydrogen gas at high temperature on a metal catalyst to form a graphene film. Graphene film growth via CVD is very appealing because large scale growth is possible and the electrical conductivity of CVD grown graphene films is generally better than that of solution processed graphene films. 

**Figure 9:**
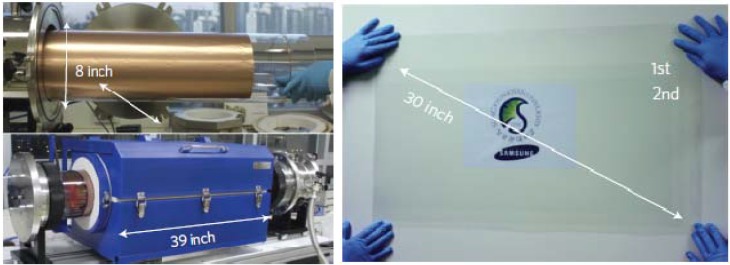
Image of a copper foil wrapped around a quartz tube to be placed in a furnace for chemical vapor deposition is shown on the left; A large area transparent graphene film transferred on a transparent substrate is shown on the right. Reprinted with permission from [23]. Copyright (2010) by the Macmillan Publishers Limited.

High quality graphene films with dimensions up to 30 in have been demonstrated by Bae *et al*. [23]. This is achieved by growing the graphene film on a thin copper foil, which is wrapped around a large quartz tube to be placed in a furnace for CVD growth, as shown in Figure 9. The graphene film grown on the copper foil can be transferred to another arbitrary substrate via transfer printing. The different variants of transfer printing and the impact on the electrical performance of the transferred graphene film will be discussed in detail in the following section (3.1.3). The graphene film grown by Bae *et al*. has also demonstrated very outstanding sheet resistance of 30 Ω/sq and 90% transmittance. 

Despite the impressive progress in the field, some challenges remain to be overcome. CVD grown graphene films that demonstrate very low sheet resistance are often doped chemically. However, chemical doping is unstable and the sheet resistance of graphene films increases with time. This will be discussed in greater detail in a subsequent section (3.2). Undoped graphene films grown via CVD do not display superb electrical properties like an exfoliated graphene film due to the presence of grain boundaries [101,102,103,104,105,106,107,108] and wrinkles [109,110,111,112]. 

Grain boundaries in graphene films have been found to decrease the local work function, leading to potential barriers that scatter charge carriers by both backscattering and intervalley carrier scattering [102]. The carrier mobility decreases and impedes electrical transport. Hence, various groups have examined methods to optimize the CVD growth in order to decrease the density of grain boundaries and increase the grain size. 

Yu *et al*. reported an approach that achieves grain size up to tens of micrometers by pre-patterning seed crystals to control the graphene nucleation [101]. In the absence of pre-patterned seed crystals, the nucleation sites form randomly and many grain boundaries can be expected.

Other groups have also adopted a similar strategy of decreasing the nucleation sites to increase the grain size and decrease the grain boundaries by various clever methods. Li *et al*. found that high temperature, low partial pressure and methane (carbon feedstock for graphene growth) flow rate during CVD growth result in fewer nucleation sites. Hence, they proposed a two-step CVD process [104]. The first step involves a high temperature and low methane flow rate and partial pressure process to generate a low density of graphene nuclei. Methane flow rate and partial pressure are increased in a subsequent step to increase the size of the graphene domain. The graphene films with larger domains that result from the two-step CVD process are shown to have high mobility due to reduced scattering at the inter-domain interfaces.

**Figure 10:**
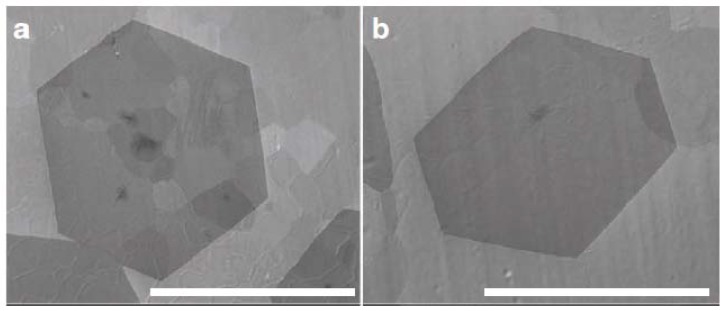
Scanning electron microscope (SEM) images of graphene grains on a Pt foil. The scale bars in are (**a**) 1mm; and (**b**) 0.5 mm respectively. Reprinted with permission from [108]. Copyright (2012) by the Macmillan Publishers Limited.

Millimeter-sized grains have been demonstrated by various groups [105,106,107,108] (Figure 10). This is achieved by a similar strategy that was mentioned earlier: initiating with a low nucleation rate and driving the nucleation sites to grow bigger in a subsequent step. In one instance, the low nucleation rate was achieved by low hydrogen flow rate and use of a polished copper catalyst substrate [105]. Polishing and pre-annealing the copper substrate reduces the defects on the substrate (e.g., impurities and surface irregularities), which can serve as nucleation centers. Higher temperature in a subsequent step drives the growth of the graphene domains to form large grains.

Besides grain boundaries, wrinkles in graphene films can impede electrical transport across the folds [110]. Wrinkles form due to thermal-induced stress during the CVD process [109,111]. High temperatures during CVD growth expands the metal catalyst film, which upon cooling, contracts. The thermal induced strain set up is accommodated by wrinkles formed in graphene films grown [111]. These wrinkles are often formed around step edges and defect lines of the substrate [109]. As mentioned earlier, polishing may be able to reduce the defect lines, which may suppress the wrinkle formation. If thin epitaxial metal films with small thermal expansion coefficients were used, the thermal induced strain and wrinkle formation are expected to decrease. The wrinkle formation is also found to be reversible by heating, which releases the strain. Hence, it may be possible to release the strain and eliminate the wrinkles during the transfer process when the graphene film is not fixed to a support [111]. The methods suggested to decrease wrinkles can potentially improve the electrical conductivity of the graphene films.

#### 3.1.3. Transfer Printing of Graphene Films

A graphene film grown via chemical vapor deposition (CVD) is often grown on a metal foil or metal film on substrate. Graphene film, which is used for transparent conductor applications has to be transfer printed from the growth substrate to a transparent substrate. Polymeric substrates are often used as they are flexible and transparent. It is not feasible to grow graphene film directly on a polymeric substrate via CVD because the polymeric substrate degrades under the high temperature employed during CVD. In this section, we discuss the various techniques developed to transfer print graphene films and relate the transfer printing process to the quality of the film transferred. 

The transfer printing process [21,23,113,114,115,116,117,118,119,120] generally involves attaching the graphene film onto a support substrate before etching the metal catalyst off. The graphene film on the support substrate is then transfer printed onto the desired receiving substrate from the support substrate. This is achieved by dissolving or peeling off the support substrate after attaching the graphene film—support substrate sandwich to the receiving substrate. 

**Figure 11:**
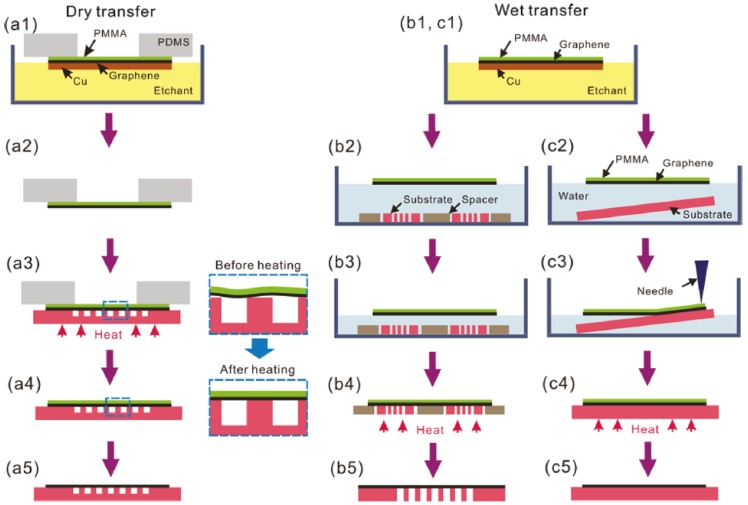
Schematic illustration of dry and wet transfer processes. (**a**) Dry transfer process onto perforated surface; (**b**) Wet transfer processes onto perforated surface and (**c**) non-perforated surface. Magnified views of (a3) and (a4) are provided. Reprinted with permission from [115]. Copyright (2011) by the American Chemical Society.

Various types of supporting substrates have been investigated. They include poly(methyl methacrylate) PMMA [114,115], polydimethylsiloxane (PDMS) [21,113], polyimide (PI) [116,117] and thermal release tape [23], of which, PMMA is one of the most commonly used supporting substrates. Suk *et al*. reported dry and wet transfer techniques using PMMA, as illustrated in Figure 11 [115]. 

In the dry transfer process, PMMA is spin-coated onto graphene film grown on copper foil. A PDMS frame was used to support the PMMA/graphene film sandwich while etching the copper foil using ammonium persulfate. This copper etchant is preferred over iron(III) nitrate, because it does not leave behind contamination residues like iron oxide. After the copper foil is completely etched, the graphene film is transferred to a receiving substrate. Heat treatment above the glass transition temperature of PMMA is performed after the graphene transfer to improve the contact between the graphene film and the receiving substrate. This is achieved by softening the PMMA so that the gap between the graphene film and the receiving substrate is reduced. The improved adhesion of the graphene film to the receiving substrate prevents cracks and tears from forming when the PMMA is removed. The wet transfer process is similar, as illustrated in Figure 11. Heat treatment is also performed to improve the quality of transferred graphene film. This method has been found to yield graphene films with lower sheet resistance. 

Liang *et al*. [114] also reported that the PMMA transfer process can be improved by performing a RCA clean, which removes any Cu or Fe residues from the copper etchant used to etch copper catalyst in graphene growth. Cracks formed in the graphene film during transfer are minimized when the adhesion of the graphene film to receiving substrate is improved. This can be achieved by increasing the hydrophilicity of the receiving substrate and baking.

The importance of adhesion between the transferred graphene film and receiving substrate is emphasized in [23]. When transfer is performed using thermal release tape, the first layer of transferred graphene film has relatively high sheet resistance (*i.e*., ~275 Ω/sq), but subsequent transfers quickly decrease the sheet resistance. Hence, it was postulated that the adhesion between the first layer of film transferred directly onto the receiving substrate and the receiving substrate is poor, which results in mechanical damage of the film when the thermal release tape is removed. Hence, the sheet resistance is poor. Subsequent transferred layers do not interact directly with the substrate, so the sheet resistance is lower.

**Figure 12:**
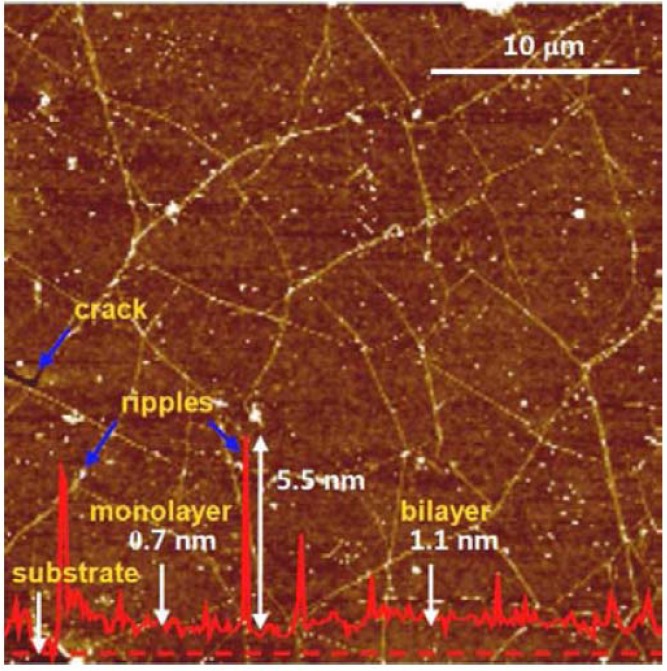
Atomic force microscope (AFM) image of a graphene film transferred onto a polyethylene terephthalate (PET) film using the thermal release tape. The solid red line is the height profile measured along the dashed red line. Reprinted with permission from [23]. Copyright (2010) by the Macmillan Publishers Limited.

It is evident that the transfer printing process of graphene films to an arbitrary receiving substrate strongly influences the quality of the transferred film. This is because the transfer process can introduce residual contaminants and mechanical damage to the transferred film (Figure 12). Hence, an optimized process to minimize the contamination and damage will be expected to enhance the quality of the transferred film and improve the electrical properties.

### 3.2. Post Treatment

**Figure 13:**
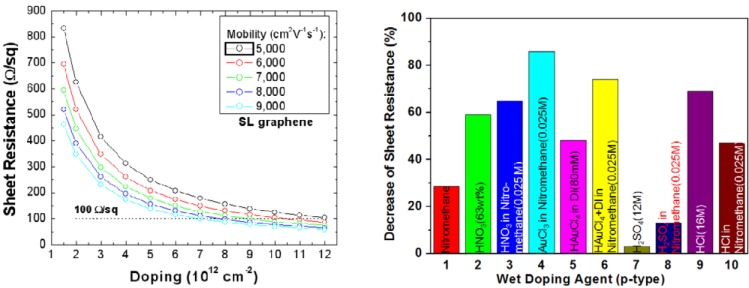
Calculated sheet resistance of a graphene film as a function of doping density with different charge carrier mobility is shown on the left. The decrease in sheet resistance using various wet doping agents is shown on the right. Reprinted with permission from [23]. Copyright (2010) by the Macmillan Publishers Limited.

Typical sheet resistance of a transferred (via PMMA) graphene film is ~125 Ω/sq. This sheet resistance is low enough for certain applications, such as touch screens. However, when integrated into a solar cell or large area display, it will not be suitable as the series resistance is too high. Wet doping agents can be used to enhance the electrical conductivity of the graphene films [23,121,122,123], as shown in Figure 13. Some typical wet chemical p-dopants include AuCl_3_ in nitromethane, HNO_3_ in nitromethane and HCl. These strong oxidizing agents withdraw electrons from the graphene film and increase the doping density, leading to a decrease in sheet resistance [23]. Although more than 80% decrease in sheet resistance has been observed in films doped by AuCl_3_ in nitromethane, the doping effect is transient. The sheet resistance increases by ~100% after 80 days in ambient condition at room temperature [121]. Another dopant studied, MoO_x_, also suffers sheet resistance degradation with time [124]. A self-assembled monolayer of fluoroalkyltrichlorosilane (FTS) is another dopant investigated [125]. However, it is not very effective in decreasing the sheet resistance (*i.e*., only 8% decrease in sheet resistance). 

Ni *et al*. reported a novel method of using nonvolatile ferroelectric polymer poly(vinylidenefluoride-co-trifluoroethylene) (P(VDF-TrFE)) gating to decrease the sheet resistance of the graphene film [126]. The advantage of the method is that electrostatic doping by the ferroelectric polymer is non-volatile so the sheet resistance stays low with time. Nonetheless, more work remains before this technology can be implemented in large-scale application. An opaque metal gate is used to apply a large electric field to polarize the ferroelectric polymer. Removal of the metal gate or an alternative way to polarize the ferroelectric polymer is essential to render transparency to the graphene-ferroelectric hybrid. 

Although single layer of chemical vapor deposition (CVD) grown graphene film has relatively low sheet resistance <500 Ω/sq, the search for a scalable, non-volatile and highly effective method to dope graphene continues so that graphene films can be used in more commercial applications [127]. The scalability, reproducibility and cost effectiveness of integrating graphene transparent conductors into practical devices also have to be carefully evaluated before successful commercialization.

## 4. Hybrid Films

Various groups have also explored hybrid films [128,129,130,131,132,133,134]. A hybrid film is desirable because it possesses the positive attributes of all its component materials. There are many variations of hybrid films, such as carbon nanotube—graphene hybrid [128,129], graphene—nanowire hybrid [130,131], graphene—metal grid hybrid [132,133] and carbon nanotube—(PEDOT) hybrid [134]. The electrical and optical properties can be improved by integrating different materials because one dimensional materials can often be used to bridge the gaps between non-uniform two dimensional materials. The selection of component materials and mixture of the component materials are critical parameters, which determine the electrical and optical properties of the hybrid films.

## 5. Conclusions

As demand for transparent conductors in flexible devices is expected to be high in future, alternative materials to substitute metal oxides are sought. Films of carbon nanomaterials have been identified as one potential class of substitute materials. More work remains as the sheet resistance of carbon nanomaterial films is still unable to reach <10 Ω/sq at 90% transparency (refer to Table 1 below), which deems them unsuitable for certain applications, such as transparent conductors in photovoltaic cells and large area displays. Better electrical and optical properties can possibly be achieved via a few means. Improved techniques to synthesize high quality carbon nanomaterials, whose properties are near those of a pristine carbon nanomaterial must be developed. Advanced methods of carbon nanomaterial film assembly or deposition, with good control of film properties and low introduction of impurities or contaminants, is essential. Better post-treatment methods to dope the films are desirable.

**Table 1:** Sheet resistance and optical transmittance of different carbon nanomaterials and their hybrids.

Material	Sheet resistance (Ω/sq)	Transmittance (%)	Reference
CNT			
CVD CNT	265	80	41
CVD CNT	265	70	42
Solution CNT	1000	80	13
Solution CNT	400	80	48
Solution CNT	100	70	63
Graphene			
CVD graphene	280	80	21
CVD graphene	30	90	23
CVD graphene	980	97.6	115
Reduced graphene oxide	8000	93	84
Reduced graphene oxide	1000	80	88
Reduced graphene oxide	1800	70	94
Hybrid			
CNT-graphene	240	85	128
Nanowire-graphene	64	94	130
Metal grid-graphene	20	90	132
CNT-PEDOT	80	75	134

Nonetheless, the films, which have been demonstrated may be suitable for less demanding applications, such as touch panels. Further exploration in their stability in devices over time is necessary. The scalability, reproducibility and cost effectiveness of integrating them into practical devices also have to be carefully evaluated. Finally, the success of practical application of carbon nanomaterial films in transparent conductors is also dependent on the development of competing alternative materials, such as thin metal films, metal nanowire films, conducting polymers and various other forms of hybrid films.
